# Sarcomatoid carcinoma associated with chronic empyema and early lung and pleural metastases: A case report

**DOI:** 10.1097/MD.0000000000025692

**Published:** 2021-05-07

**Authors:** Jeong keyom Kim, Min Seon Kim, Kyung Hee Lee, Ro Woon Lee, Lucia Kim

**Affiliations:** a Department of Radiology; b Department of Pathology, Inha University Hospital, Inha University College of Medicine, Jung-gu, Incheon, South Korea.

**Keywords:** chronic empyema associated malignancy, computed tomography, metastasis, sarcomatoid carcinoma

## Abstract

**Introduction:**

The relationship between chronic empyema and malignant tumors, most of which are lymphoma, has been recognized for many decades. Sarcomatoid carcinoma associated with chronic empyema is extremely rare, may metastasize to other organs in the early stage, and rapidly progresses to death. As far as we know, this was the first case report on sarcomatoid carcinoma associated chronic empyema.

**The patient's main concerns and important clinical findings:**

A 59-year-old man presented to our hospital with a 9-year history of chronic empyema and a chief complaint of left chest wall pain for 5 months. The diagnostic contrast-enhanced computed tomography (CT) showed a large irregular soft tissue mass located on the left lower hemithorax at the margin of the empyema cavity extending to the adjacent chest wall and lung parenchyma. In addition, CT revealed pleural and pulmonary metastases surrounded by ground glass opacity.

**The main diagnosis, therapeutics interventions, and outcomes:**

The patient underwent CT guided percutaneous core needle biopsy (PCNB). The histopathological evaluation showed carcinomatous proliferation of pleomorphic spindle cells with extensive necrosis. Immunohistochemically, tumor cells were positive for cytokeratin and vimentin. The final histopathological diagnosis was sarcomatoid carcinoma underlying chronic empyema. The tumors showed rapid progression on serial simple radiography. Palliative treatments were performed, but the patient still developed severe dyspnea and died shortly after on day 16.

**Conclusion:**

Sarcomatoid carcinoma can occur very rarely as a complication of chronic empyema, and is more aggressive than usual. Early detection of developing malignancy during the follow-up of chronic empyema is an important factor for patient prognosis.

## 1 Introduction

Sarcomatoid carcinoma is a poorly differentiated carcinoma that exhibits histological characteristics of both malignant epithelial and mesenchymal cells.^[1–3]^ These malignancies can occur anywhere in the body, but rarely occur in the pleura.^[4,5]^ Chronic empyema is widely known as one of the disposing factors of malignancy, and a majority of these tumors are lymphomas.^[1]^

Here, we present an extremely rare case of chronic empyema-associated sarcomatoid carcinoma with hypervascular lung and pleural metastasis. To the best of our knowledge, there has never been a reported case in the English literature to date of a patient with sarcomatoid carcinoma associated with chronic empyema. We describe the imaging findings of chronic empyema-associated sarcomatoid carcinoma, which can potentially aid in early detection and diagnosis of future cases.

## 2 Case presentation

A 59-year-old man presented to our hospital with a chief complaint of left chest wall pain for 5 months. He had a 12-year history of hemodialysis in the Department of Renal Medicine of our hospital for end-stage renal disease due to immunoglobulin A nephropathy (IgA nephropathy) and a history of renal cell carcinoma (RCC) treated with radical nephrectomy 2 years prior. Nine years ago, he was hospitalized for community-acquired pneumonia with parapneumonic effusion, which subsequently developed to chronic empyema. During the follow-up period, the empyema remained stable and the patient had no symptoms. Therefore, surgical intervention, such as decortication, was not performed. Physical examination revealed no other clinical signs or symptoms, and there was no history of chest wall trauma. His vital signs were within the normal range (body temperature 36.5 °C, pulse rate 60 beats/min, blood pressure 120/80 mmHg, and respiratory rate 12 breaths/min). Laboratory examinations revealed mild anemia, with a hemoglobin level of 9.5 g/dL (normal range, 13.1–17.5 g/dL) and an increased C-reactive protein level (6.22 mg/dL). Other routine blood counts and biochemistry were normal, with negative urinalysis findings.

**Figure 1:**
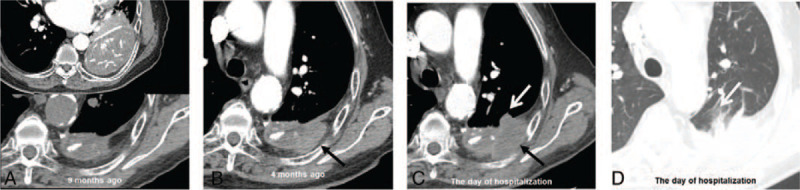
Computed tomography (CT) of the chest at different times. (A) Contrast enhanced CT scan showed a chronic empyema cavity with a peripheral rim and dystrophic calcifications in the left lower hemithorax. (B) Chest CT images taken 4 months ago showed a lenticular mass (black arrow) at the edge of the chronic empyema (3.7 cm in size) which was newly occurring compared with prior CT. (C, D) Follow-up chest CT images taken at admission showed a remarkable enlargement of the prior pleural soft tissue mass with extension to the adjacent chest wall (black arrow) and invasion of the lung parenchyma (white arrow).

**Figure 2:**
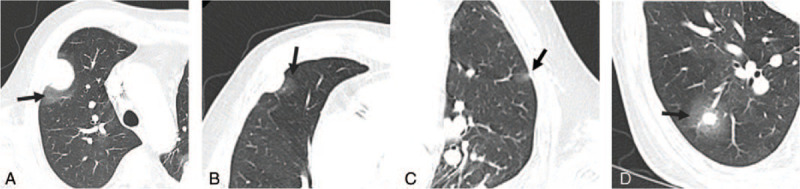
Computed tomography (CT) of the chest at admission. (A–D) The lung setting images showed multiple pleural metastases in the bilateral hemithorax and pulmonary metastasis in right lower lobe. The lung setting images demonstrated that each nodule was surrounded by a ground glass opacity with a halo sign (black arrow) representing hypervascular metastases.

The contrast-enhanced thoracic computed tomography (CT) scan showed an empyema cavity enclosed by calcified pleural layers in the left lower hemithorax (Fig. 1A) and an approximately 5.2 × 3.2 cm sized irregular soft tissue mass located on the posterior pleura eccentrically at the margin of empyema cavity (Fig. 1C). The mass extended to the adjacent chest wall and lung parenchyma with perilesional ground glass opacity (Fig. 1C, D). Prior thoracic CT performed 4 months ago, which was done for regular follow-up of chronic empyema, demonstrated lenticular soft tissue involving extrapleural fat (Fig. 1B) in the corresponding area. It was thought that the mass was overlooked at the time because the attenuation of the empyema cavity and the tumor were similar. Subsequently, there were multiple pleural and lung metastases on diagnostic CT, which were not seen in previous CT. Each mass or nodule showed a surrounding halo of ground glass opacity (Fig. 2A–D). These CT findings are suggestive of hypervascular metastases. The tumor appeared to be rapidly growing and highly aggressive. Our initial diagnosis is RCC metastases because of the patient's medical history of RCC and RCC usually leads to hypervascular metastases. However, since the primary mass initially arose from the margin of the chronic empyema, highly aggressive chronic empyema associated malignancy (CEAM) such as sarcoma could not be excluded.

**Figure 3:**
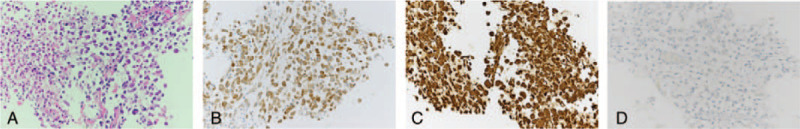
(A) Microscopically, the tumor consisted mostly of slightly scattered phleomorphic cells and extensive necrosis with a few spindle cells (HE, magnification 200×). Immunohistochemically, the tumor cells were positive for cytokeratin (B) and vimentin (C), but negative for PAX8 (D). PAX8 = paired box gene 8.

The patient underwent CT-guided percutaneous core needle biopsy (PCNB). Biopsy was performed in the pleural mass of the right upper hemithorax because the margin of the primary tumor of the left lower hemithorax was not well distinguished from the chronic empyema cavity on CT. Histopathological evaluation revealed carcinomatous proliferation of pleomorphic spindle and globoid cells with extensive necrosis (Fig. 3A). Immunohistochemically, tumor cells were positive for cytokeratin and vimentin, but negative for cytokeratin 7 (CK 7), thyroid transcription factor-1 (TTF-1), carbonic anhydrase-9 (CA-9), paired box gene 8 (PAX8), leukocyte common antigen (LCA), and human melanoma black 45 (HMB45) (Fig. 3B–D). The final histopathological diagnosis was sarcomatoid carcinoma.

**Figure 4:**
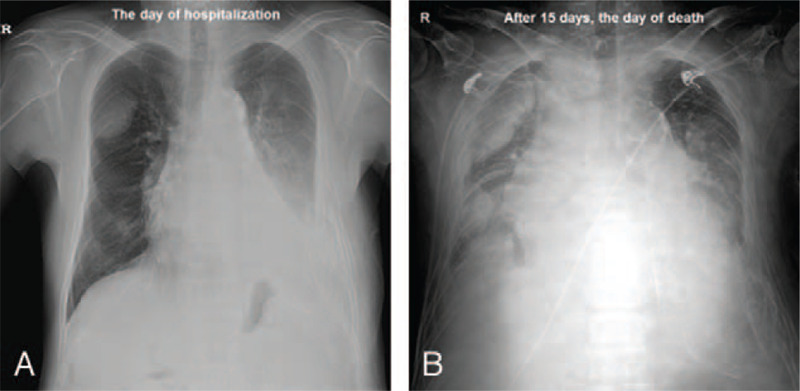
Serial simple radiography showed rapid progression of the tumor in the right hemithorax and pulmonary edema, when comparing images performed on the day of hospitalization (A) and the day of death (B).

After admission, the patient complained of severe dyspnea and showed rapid clinical deterioration with a progressively decreased level of hemoglobin. Serial simple radiography showed rapid progression of the tumors (Fig. 4A, B). Patient was started on oxygen therapy and palliative treatment for symptom control. Nevertheless, on hospital day 16, after 9 days from biopsy, the patient expired.

## 3 Discussion

Sarcomatoid carcinoma is a rare, aggressive, and malignant cancer composed of both epithelial and non-epithelial sarcomatous components.^[4]^ Primary sarcomatoid carcinoma predominantly affects elderly patients and can occur in various anatomical sites.^[4,5]^ Primary sarcomatoid carcinoma of the pleural is extremely rare, and only a few cases have been reported in the literature.^[6]^

Sarcomatoid carcinomas have a strong male predominance and are associated with heavy smoking.^[4]^ Few studies have suggested that patients with sarcomatoid carcinoma are exposed to asbestos.^[5]^ The etiology of this tumor remains unclear, but chronic inflammation and toxicity are possible direct mechanisms of the development of sarcomatoid carcinoma.^[7]^ No studies have suggested a possible association between chronic empyema and sarcomatoid carcinoma. CEAM is a well-known complication of long-standing empyema and is not extremely rare.^[1–3,6,8]^ The prevalence of CEAM has been reported to vary from 2.67% to 5.2%.^[2,6]^ The most common malignancies are non-Hodgkin lymphoma and various other malignancies such as squamous cell carcinoma, mesothelioma, malignant fibrous histiocytoma, liposarcoma, rhabdomyosarcoma, angiosarcoma, and hemangioendothelioma.^[1]^ CEAM has been reported in East Asia, including Japan, which is associated with a high prevalence of tuberculosis and active therapy with artificial pneumothorax and thoracoplasty.^[1,3,6,9]^ It is relatively rare that malignancy occurs in a patient like ours with chronic empyema caused by CAP who has never received surgical treatment.^[1]^ Perhaps, a long-standing chronic inflammation due to chronic empyema may be the causative mechanism that stimulates mesothelial cells or activates oncogenic substances contained in the pleura, and can lead to immunocompromised conditions.^[7]^

The clinical manifestations of sarcomatoid carcinoma are not specific and can differ according to their locations. Sarcomatoid carcinoma of the pleura usually presents with the nonspecific finding of pleural soft tissue masses on CT, and a diagnostic challenge on imaging.^[1]^ However, chronic empyema-associated sarcomatoid carcinoma in our patient showed imaging features similar to those of other CEAMs. In our case, CT demonstrated a lenticular soft tissue mass around the calcified empyema cavity and invading the adjacent extrapulmonary fat, which suggested malignancy occurred in association with the chronic empyema. The attenuation of the mass and the empyema cavity is similar, so malignancy can be missed if care is not taken. It can be difficult to distinguish sarcomatoid carcinoma from other CEAMs or long-term complications of chronic empyema such as hemorrhage on CT.^[3,6]^

Metastases of pleural sarcomatoid neoplasms occur mainly in the lung parenchyma and mediastinal lymph nodes, but it can also occur in extra-thoracic sites such as the liver, bones, peritoneum, and adrenal glands.^[4]^ In our case, CT demonstrated aggressive pleural and pulmonary metastases exhibiting a halo sign. The halo sign is a CT finding of ground-glass opacity surrounding a nodule or mass that suggests the hypervascular nature of the metastatic nodule. Hypervascular metastasis is more common in angiosarcoma, choriocarcinoma, and renal cell carcinoma, and has rarely been reported in sarcomatoid carcinoma.^[10]^ Therefore, RCC was first considered in our patient's case, but CEAM with sarcoma components can show hypervascular metastasis and should be considered for differential diagnosis.

The treatment plan for pleural sarcomatoid carcinoma involves surgical resection or chemotherapy depending on the stage of the disease.^[8]^ Overall, the prognosis of sarcomatoid neoplasms of the lung and pleura is poor. Because sarcomatoid carcinoma of the pleura is extremely rare, the median survival rate is not well known. The median survival period for patients with pulmonary sarcomatoid carcinoma is known to be 9.9 months.^[8]^ In our case, the tumor showed very rapid progression, and the patient expired only 5 months after the primary mass was first seen on CT. Therefore, our case suggests that early detection is very important for the patient's prognosis when CEAM has a sarcomatoid component.

## Acknowledgments

The study sponsor had no involvement in the conduct of the study or in the writing of the article.

## Author contributions

**Data curation:** Jeong Kyeom Kim, Min Seon Kim, Lucia Kim.

**Supervision:** Kyung Hee Lee.

**Writing – original draft:** Jeong Kyeom Kim, Min Seon Kim.

**Writing – review & editing:** Jeong Kyeom Kim, Min Seon Kim, Ro Woon Lee.
